# Development of an Interactive Global Surgery Course for Interdisciplinary Learners

**DOI:** 10.5334/aogh.3178

**Published:** 2021-03-31

**Authors:** Tamara N. Fitzgerald, Nyagetuba J. K. Muma, John A. Gallis, Grey Reavis, Alvan Ukachukwu, Emily R. Smith, Osondu Ogbuoji, Henry E. Rice

**Affiliations:** 1 Department of Surgery, Duke University, Durham, NC, USA; 2 Duke Global Health Institute, Durham, NC, USA; 3 Department of Surgery, AIC Kijabe Hospital, Kijabe, Kenya; 4 Learning Innovation, Duke University, Durham, NC, USA; 5 Department of Surgery, Asokoro District Hospital, Abuja, Nigeria; 6 Department of Public Health, Baylor University, Waco, TX, USA; 7 Center for Policy Impact in Global Health, Duke Global Health Institute, Durham, NC, USA

## Abstract

**Introduction:**

Global surgical care is increasingly recognized in the global health agenda and requires multidisciplinary engagement. Despite high interest among medical students, residents and other learners, many surgical faculty and health experts remain uniformed about global surgical care.

**Methods:**

We have operated an interdisciplinary graduate-level course in Global Surgical Care based on didactics and interactive group learning. Students completed a pre- and post-course survey regarding their learning experiences and results were analyzed using the Wilcoxon signed-rank test.

**Results:**

Fourteen students completed the pre-course survey, and 11 completed the post-course survey. Eleven students (79%) were enrolled in a Master’s degree program in global health, with eight students (57%) planning to attend medical school. The median ranking of surgery on the global health agenda was fifth at the beginning of the course and third at the conclusion (p = 0.11). Non-infectious disease priorities tended to stay the same or increase in rank from pre- to post-course. Infectious disease priorities tended to decrease in rank (HIV/AIDS, p = 0.07; malaria, p = 0.02; neglected infectious disease, p = 0.3). Students reported that their understanding of global health (p = 0.03), global surgery (p = 0.001) and challenges faced by the underserved (p = 0.03) improved during the course. When asked if surgery was an indispensable part of healthcare, before the course 64% of students strongly agreed, while after the course 91% of students strongly agreed (p = 0.3). Students reported that the interactive nature of the course strengthened their skills in collaborative problem-solving.

**Conclusions:**

We describe an interdisciplinary global surgery course that integrates didactics with team-based projects. Students appeared to learn core topics and held a different view of global surgery after the course. Similar courses in global surgery can educate clinicians and other stakeholders about strategies for building healthy surgical systems worldwide.

## Introduction

Recent publications have highlighted the need for surgical system strengthening in low- and middle-income countries (LMICs) and for increased advocacy to advance global surgical care [1]. Due to efforts from many stakeholders, interest and awareness of global surgery is steadily increasing in both academic surgery and the global health arena [2,3]. In particular, medical students and residents have demonstrated an increasing desire for global surgery experiences and education. With few formalized educational opportunities [4], students and surgical faculty remain relatively misinformed about global surgical care [2,5].

Previous reports of academic classes specific to global surgery are limited, with most experiences describing short-term (days to weeks) courses taught in high-income countries (HICs) designed for clinical learners in those settings. Several clinical training programs in North America have described formalized clinical surgical rotations with partner institutions in LMICs [6], while others have integrated a short-term didactic curriculum to prepare medical students for a surgical rotation in a LMIC [7,8]. Several North American surgical residency programs are beginning to offer a global surgery tract to prepare residents for a career in global surgery [9]. In addition to clinical and public health training, there has been a call for development of an ethics curriculum specific to global surgery, as complex ethical challenges have been described [10]. There are currently no descriptions in the literature of comprehensive courses on global surgery that incorporate interdisciplinary skills. It is crucial that education strategies be developed to ensure that the next generation of global researchers, clinicians and educators understand the challenges of this field and can develop innovative solutions to global surgical challenges. In the future, learners and faculty should be comprised of diverse country origins, institutions and socioeconomic backgrounds.

In this paper we describe the development and evaluation of a global surgery course developed for an interdisciplinary audience of learners. This course was designed based on a structured interdisciplinary framework for several reasons. First and foremost, global surgery necessitates collaboration between multiple disciplines including clinical areas (surgery, anesthesia, nursing, obstetrics, midwifery, critical care, etc.), biomedical engineering, public health, economics, advocacy and policy development and others [11,12]. Secondly, global surgery has been referred to as the “neglected stepchild of global health” [13]. To bring surgery to the forefront, we must educate our colleagues and students in global health about the importance of surgical care as an integral component of functional health systems and within the global health agenda. The goals of this report are to describe the course, and to summarize its impact on student knowledge and views of global surgery.

## Methods

### Setting

The Global Surgical Care course was developed through the Duke University School of Medicine Department of Surgery and the Duke Global Health Institute (DGHI). The DGHI is a multidisciplinary institution that includes faculty working in partnership with colleagues in over 40 countries, including Duke faculty from the Schools of Arts and Sciences, Divinity, Engineering, Environment, Law, Public Policy, Medicine and Nursing. Over 400 students per year are enrolled in undergraduate and graduate programs within the DGHI. The Global Surgical Care course has been approved through the DGHI as a “Methods in Global Health Course,” acknowledging that it provides learners with training in research methods specific to global health.

### Curriculum Design and Course Development

The curriculum is designed as a semester-long seminar with didactic sessions, class discussions and reading of peer-reviewed publications to focus on the topics set forth in the Lancet Commission on Global Surgery [1]. Major topics include: Burden of Disease, Surgical Infrastructure, Innovation and Biomedical Engineering, Surgical Workforce, Economics, Health Financing and National Surgical Planning. The course has been taught in this format annually since 2016. Most lectures have been given by guest speakers from Duke University as well as our partners around the world with topical expertise (***Table 1***). As the course progressed, we have been able to incorporate more lecturers from LMICs, using internet-based communication platforms, with a question and answer period to follow.

**Table 1:** Guest lecturers who contributed to the Global Surgical Care Course.


Emanuel Ameh Professor and Chief Consultant Pediatric Surgeon National Hospital Abuja, Nigeria	Gita Mody Assistant Professor of Thoracic Surgery University of North Carolina at Chapel Hill Chapel Hill NC, USA

Anirudh Krishna Professor of Public Policy and Political Science Duke University Durham NC, USA	Michael Penn Director of Communications Duke Global Health Institute Durham NC, USA

Nyagetuba J K Muma Consultant Pediatric Surgeon Executive Director AIC Kijabe Hospital Kijabe, Kenya	Abdullah Saleh Director of Office of Global Surgery University of Alberta Edmonton, Canada

Alvan Ukachukwu Consultant Neurosurgeon Asokoro District Hospital Abuja, Nigeria	Emily Smith Assistant Professor of Epidemiology Baylor University Waco TX, USA

Osondu Ogbuoji Assistant Research Professor Deputy Director Center for Policy Impact in Global Health Duke Global Health Institute Durham NC, USA	Lubna Samad Consultant Pediatric Surgeon Aga Khan University Karachi, Pakistan

Tolu Oladele Consultant Obstetrician & Gynecologist Assistant Director Health Sector Response Support Division National Agency for the Control of AIDS Abuja, Nigeria	Ann Saterbak Professor of Biomedical Engineering Duke University Durham NC, USA

Robert Malkin Professor of Biomedical Engineering Duke University Durham NC, USA	Robert Ssekitoleko Lecturer in Biomedical Engineering Makerere University Kampala, Uganda


**Figure 1:**
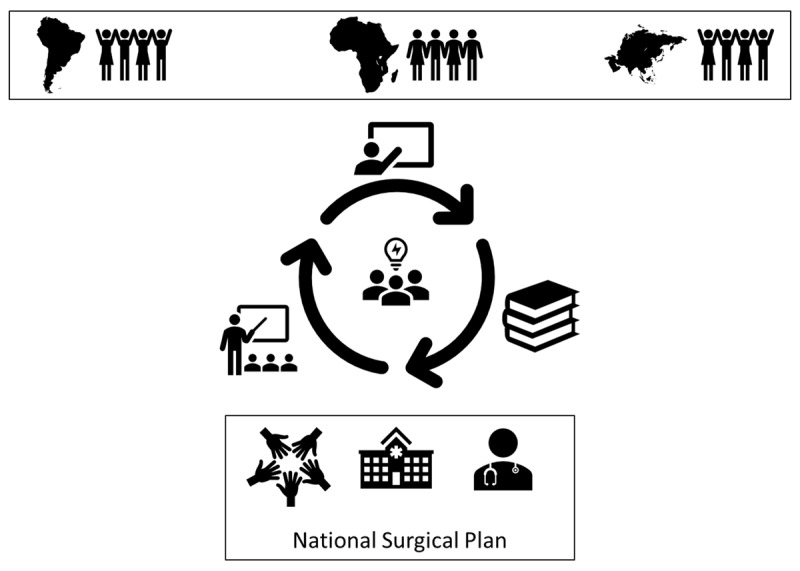
Diagram of the course structure. Students work in teams, based on their world region of interest. Each team chooses a LMIC country where they will focus their attention for the remainder of the course. During the course, new material is introduced in lectures and readings. Students are expected to apply what they have learned to a real-world assignment, focusing on surgical research and capacity building in LMICs. Upon return to class, the students teach the class what they have learned and share the research methods they have designed. By completing the readings and assignments, students have the necessary tools to draft a national surgical plan for a LMIC as their final project.

In 2019, the course was modified to encourage more interaction and to inspire students to apply the knowledge learned in class to real-world challenges. Additional topics such as Ethics in Global Surgery and Safety and Quality Initiatives have been added to the curriculum. ***Figure 1*** illustrates the structure and flow of the course. The current format is structured around three concepts: team-based learning, “flipped classroom” approach, and National Surgical, Obstetrical, Anesthesia Planning (NSOAP).

#### 1. Team-based learning

On the first day of class, students are required to complete a questionnaire regarding the region of the world in which they are most interested. Students are divided into teams of 3-4 people according to their geographic region of interest. The teams typically choose one LMIC from that region, on which they will focus their assignments for the remainder of the course. Infrequently teams have chosen to focus on a population within a HIC that struggles with access to surgical care. Most assignments are designed to be completed in teams, but there are also individual assignments.

#### 2. “Flipped Classroom” Approach

The format of the course is illustrated in ***Table 2***. The course meets 2.5 hours weekly for 14 weeks. For the first hour, students present the assignment from the previous week, acting as instructors in a “flipped classroom” approach. The assignment requires that they complete the course readings, synthesize the previous lecture material and apply this knowledge to their chosen LMIC. Often the assignment will require students to conduct research on what is known in their country regarding the topic, and then design a study to evaluate what is unknown. Students prepare a presentation to teach the class what they have learned on the topic as it pertains to their chosen LMIC. They are graded both on the quality of the presentation and their participation in discussion. The last hour of the course consists of a lecture by the course instructors or guest speakers on a new topic and introduces the assignment for the next week. Guest speakers are often incorporated into the class through internet-based teleconferencing, allowing speakers from distant locations to interact with the students in real time.

**Table 2:** Course Lectures, Assignment and Readings.


WEEK	LECTURE TOPIC	ASSIGNMENT FOR NEXT WEEK	READINGS

1	**Lancet 5 key messages**	Find a recently published media piece about global surgery and discuss what message is conveyed.	[13,28]

2	**Burden of Surgical Disease**	Using the Burden of Disease website [29], conduct a conversation with 5 lay persons regarding how the burden of disease has changed over time in your LMIC.	[30,31,32]

3	**Infrastructure**	Design a survey study to assess surgical infrastructure in your chosen LMIC.	[33,34,35]

4	**Safety and Quality**	Design a Safety & Quality Improvement Project for a global surgery issue relevant to your chosen LMIC.	[36,37]

5	**Ethics**	Prepare an ethical debate on a topic relevant to global surgery in your chosen LMIC.	[38]

6	Midterm – Design a media piece to advocate for a global surgery issue in your chosen LMIC.		

7	**Innovation & BME**	Design a feasibility study to assess a medical device developed for use in LMICs.	[39,40,41,42]

8	**Surgical Workforce: Metrics & Task Shifting**	Design a Surgical Workforce Assessment to be conducted in your chosen LMIC.	[43,44]

9	**Surgical Workforce: Education**	Develop a surgical education initiative for your chosen LMIC and describe how you will assess its efficacy.	[45,46]

10	**Macroeconomics**	Conduct an economic analysis for your chosen LMIC.	[47,48]

11	**Microeconomics**	Design a survey to assess economic impact of a surgical condition to the patient’s family in your chosen LMIC.	[7,49,50]

12	**Financing**	Final Project:	[51,52,53]

13	**National Surgical Planning**	Develop a national surgical plan for your chosen LMIC.	[54,55,56,57,58]

14	Final Exam – Presentations of National Surgical Plans.		


#### 3. Development of a NSOAP

With each assignment, students are learning methods for research in global surgery, but they are also collecting data as it pertains to the components of global surgery in their chosen LMIC. For example, over the course of the semester students will collect data regarding the current surgical infrastructure, ethical dilemmas, metrics on surgical capacity, presence of surgical education programs, and economic data for their country of interest. For the final project, students are required to integrate this research into development of a national surgical plan for their chosen LMIC.

#### 4. Feedback and Assessment

To assess changes in student knowledge and views of global surgery, we used a pre-post survey to compare global health priorities for a single course (2019). The study was approved by the Institutional Review Board at Duke University. Students were given time during the first and final class periods to complete an anonymous survey, with all results summarized at a class level. Study data were collected and managed using Qualtrics, a secure online data collection system. A research coordinator not associated with the course obtained informed consent from students to use their survey data, and then tabulated the results in an anonymized fashion. Therefore, the instructors were blinded to which students had chosen to participate, and participation in the survey did not influence students’ grades.

The survey contained demographics and questions focused on intended career choices, knowledge and attitudes towards global surgery and other areas of global health (Appendix A). Likert scale questions were used. Additionally, students were asked to rank priorities in global health before and after the course. Open-ended responses were analyzed by two of the authors using the constant comparative method [14,15], which requires categorization of quotes from participants in an iterative fashion to identify recurring themes.

Descriptive statistics were generated for demographics, as well as for both pre- and post-course survey results. Survey results were compared pre- and post-course using Wilcoxon signed-rank test. The statistical analysis was performed in R (version 3.5.0) and P values are reported.

## Results

During the study period (Fall 2019), there were 15 students enrolled in the course. Fourteen consented to allow use of their survey data and completed the pre-course survey, yielding a response rate of 93% (***Table 3***). Eleven students completed the post-course survey. There were eight female participants (57%), with most participants pursuing a Master’s degree in global health (n = 11, 79%). There were two undergraduates majoring in global health (14%) and one doctoral student from another field of study (7%). Prior to the course, the future goals of participants included medical school (n = 8, 57%), law school (n = 1, 7%), working as a research analyst for a non-governmental organization (NGO) (n = 1,7%), program management for an NGO (n = 1, 7%), academic global health research (n = 1, 7%) and undecided (n = 2, 14%). At the conclusion of the course, one student moved from wanting to pursue a research analyst position at an NGO to academic global health research, one student moved from pursuing medical school to another professional school, and one student moved from pursuing program management at an NGO to another professional school.

**Table 3:** Demographics of Participants.


	PRE-COURSE N = 14 (%)	POST-COURSE N = 11 (%)

**Gender**		

Male	6 (43%)	

Female	8 (57%)	

**Current Academic Pursuit**		

Master’s in global health	11 (79%)	

Undergraduate major in global health	2 (14%)	

Ph.D.	1 (7%)	

**Future Goals**		

Medical school	8 (57%)	5 (36%)

Law School	1 (7%)	1 (7%)

Research analyst for NGO	1 (7%)	0

Program management for NGO	1 (7%)	0

Global Health Academic Research	1 (7%)	2 (14%)

Other professional school	0	2 (14%)

Undecided	2 (14%)	1 (7%)

**Future Focus in Global Health**		

Health Systems Strengthening	2 (14%)	2 (14%)

Cancer	2 (14%)	1 (7%)

Surgery	2 (14%)	1 (7%)

Infectious disease	2 (14%)	2 (14%)

Maternal child health	2 (14%)	1 (7%)

Non-communicable diseases	1 (7%)	2 (14%)

Immigration	1 (7%)	1 (7%)

Mental health	0	1 (7%)

Undecided	2 (14%)	0


### Global Health Priorities

***Table 3*** shows the students’ focus areas in global health. Prior to the course students were interested in health systems strengthening (n = 2, 14%), cancer (n = 2, 14%), global surgery (n = 2, 14%), infectious disease (n = 2, 14%), maternal child health (n = 2, 14%), non-communicable disease (n = 1, 7%), immigration/refugee health (n = 1, 7%) and two were undecided (14%). At the conclusion of the course, one undecided student chose infectious disease and one undecided student chose health systems strengthening. One student initially interested in maternal child health changed their focus to global mental health and one student interested in neglected infectious disease changed their focus to non-communicable disease. The remaining students kept their original focus.

**Figure 2:**
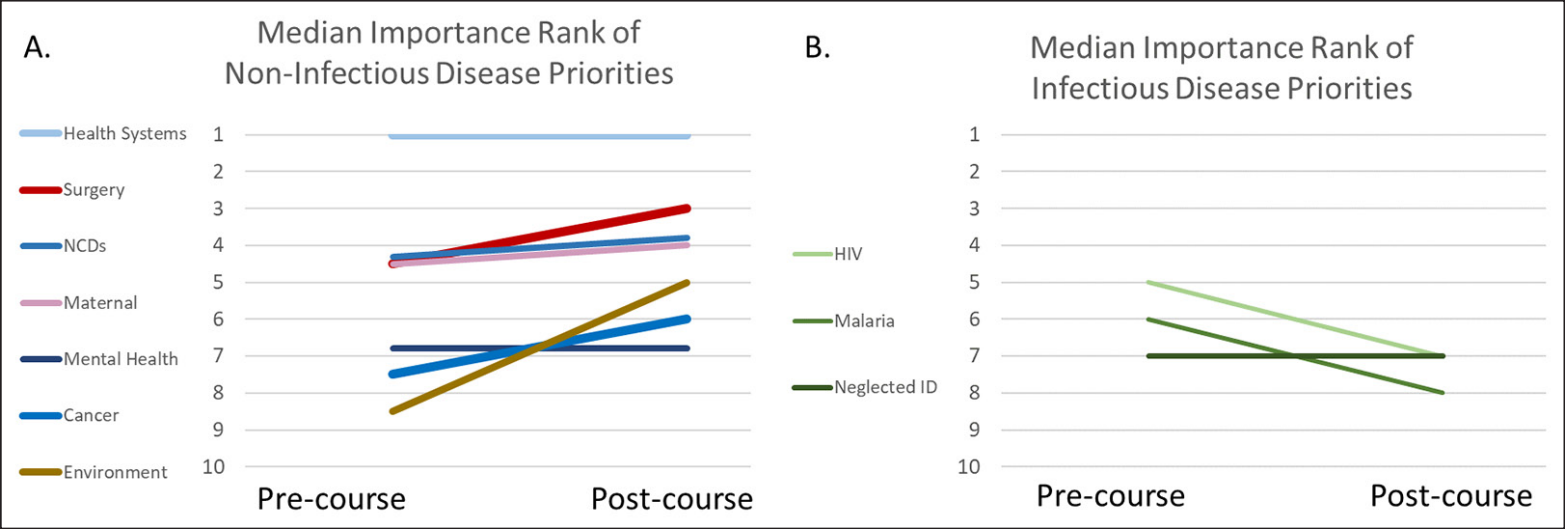
Median student ranking of global health priorities. **A)** The median importance rank for non-infectious disease priorities tended to increase during the course. Surgical care before the course was ranked on average as the fourth or fifth priority and after the course was ranked as the third priority in global health. **B)** The median importance rank for infectious disease priorities tended to decrease during the course.

Students were asked to rank a list of global health priorities before and after the course, with #1 being the most important (***Figure 2***). The pre-course median ranking of surgery was fifth on the list, and then third at the conclusion of the course (p = 0.11). Throughout the course, all but three students ranked health systems strengthening as the #1 priority in global health. ***Figure 2*** shows that non-infectious disease priorities tended to stay the same or increase in rank from pre- to post-course, although the results were not statistically significant (maternal child health p = 0.41, cancer p = 0.29, environment p = 0.25). Infectious disease priorities tended to decrease in rank importance (HIV/AIDS p = 0.07, malaria p = 0.02, neglected infectious disease p = 0.3).

### Benefits of the Course, Interactive Assignments and Group Dynamics

**Figure 3:**
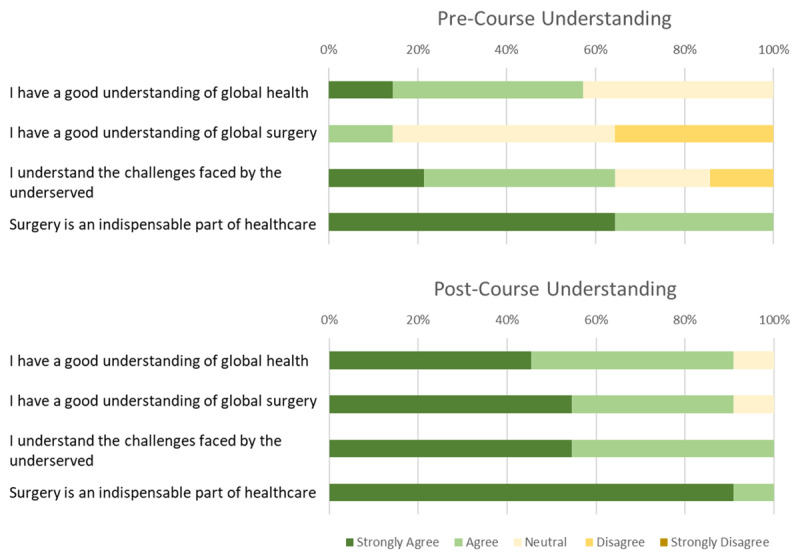
Student self-perceived understanding of global health topics before and after the course. Students felt that their understanding of global health, global surgery and the challenges faced by the underserved had improved during the course. At the conclusion of the course, 93% of students strongly agreed that surgery is an indispensable part of healthcare.

Comparison of pre-course versus post-course understanding of global health topics showed that students reported an improved understanding of global health (p = 0.03), global surgery (p = 0.001) and challenges faced by the underserved (p = 0.03) (***Figure 3***). When asked if surgery was an indispensable part of healthcare, before the course 64% of students strongly agreed that it was, while after the course 91% of students strongly agreed (p = 0.3).

**Figure 4:**
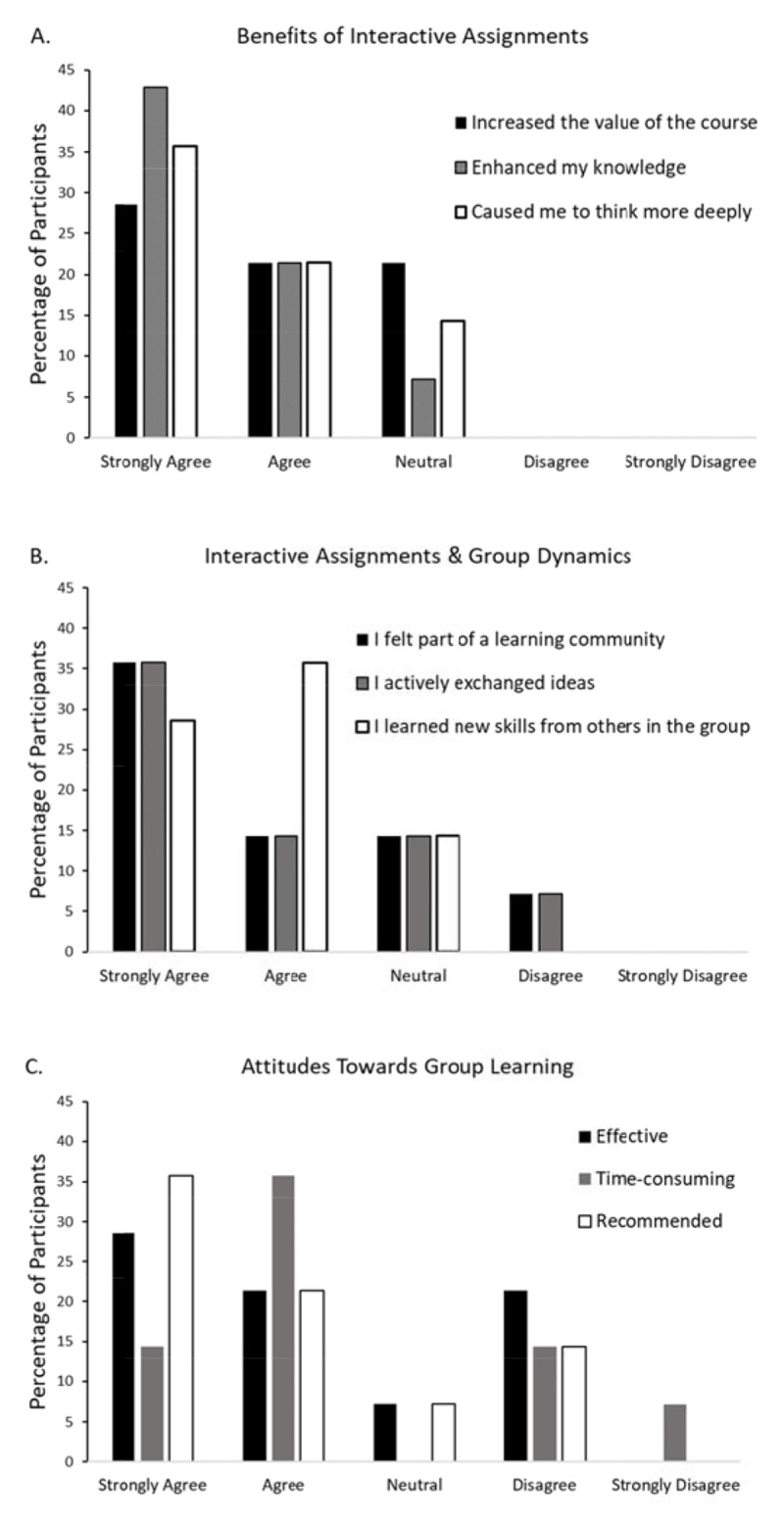
Student reflections on interactive assignments and group learning. **A)** Most students agreed that interactive assignments increased the value of the course, enhanced their knowledge of the course material and caused them to think more deeply about the concepts presented in the course. **B)** Most students reported that they learned new skills from others in the group, but there were mixed experiences regarding feeling a part of a learning community and being able to actively exchange ideas. **C)** Students had mixed experiences regarding if group learning was effective, time-consuming, or recommended.

Most students (n = 7, 64%) felt that interactive assignments increased the value of the course, enhanced their knowledge of global surgery (n = 9, 82%) and caused them to think more deeply about the topics (n = 8, 73%) (***Figure 4A***). However, the impact of group dynamics was more variable (***Figure 4B***). Three students (27%) felt that they were not part of a learning community, three students (27%) felt they did not actively exchange ideas in their group and two students (18%) did not feel that they learned skills from others in the group. Most students felt that group learning was effective (n = 7, 64%) and recommended (n = 8, 73%), but also more time-consuming (n = 7, 64%) (***Figure 4C***).

***Table 4*** displays the themes that were identified in free responses. Students commented that the group assignments fostered discussion, ideas exchange and learning from another (n = 11, 100%), and collaboration drew attention to aspects of the course material that may have been overlooked on an individual basis (n = 3, 27%). Two students (18%) commented that they learned something new about themselves by working in a group. When individuals did not contribute equally to the group then this was a source of stress (n = 9, 82%) and students found it difficult to coordinate their schedules to work on assignments outside of class (n = 4, 36%). Five students (45%) felt that the assignments were too time-consuming. Specific examples of student comments include the following:

- “We got to know ourselves better and had effective exchange of ideas and opinions. Collaboration helped draw attention to some area of the course material that one ordinarily overlooked.”
- “Our group was particularly engaged with the material as we had a member with real-life experiences which took the material out of classroom context and made it more applicable to real world issues.”
- “More than adding value to the topical understanding, working collaboratively helped me find my way around pragmatic issues of understanding what my peers desired from the material, what were our shared ambitions and divided opinions, and how should I balance wanting to ‘do good work’ with enjoying doing it.”
- “The multiple lecturers were useful to show how all backgrounds work together on global surgery topics.”

**Table 4:** Themes identified in free responses regarding students’ attitudes towards the course.


THEMES	N = 11 (%)

Group assignments fostered discussion/ideas exchange/learning from one another.	11 (100%)

Individuals did not contribute equally to group work and that was a source of stress.	9 (82%)

Assignments were time-consuming.	5 (45%)

Coordinating schedules to work in groups was difficult.	4 (36%)

Collaboration drew attention to aspects of the course material that I may have overlooked on my own.	3 (27%)

I learned something about myself from working in a group.	2 (18%)

Having diverse lecturers was a strength of the course.	2 (18%)

Working in a group made it easier to distribute the work.	2 (18%)

Wanted more class discussion of the readings.	2 (18%)

Wanted to study more countries.	1 (9%)

Group learning made more effort to compile the work and come to agreement.	1 (9%)

The assignments made the material more applicable to real life.	1 (9%)


## Discussion

In this paper we describe an interactive and interdisciplinary course designed to teach participants the fundamental components of global surgical care and research methods to advance the science of global surgery. This course utilizes several unique components, which we envision to elevate the status of academic global surgery and offer a platform to educate clinical and non-clinical trainees about the role of surgery within the global health agenda. We believe that global surgical education should grow to include a universal framework, in which contributors from LMICs and HICs collaborate to develop a comprehensive curriculum. While we have yet to achieve that level of breadth in this course, we submit our pilot experience as a starting point for consideration in design of future education initiatives.

### Educational Initiatives in Global Surgery

Many reports have shown that collaborative efforts between HIC and LMIC surgical programs have the potential to educate and benefit learners in both settings [4,6,7,16,17,18,19,20,21,22,23,24]. There is a growing interest among trainees in HICs for education opportunities in global surgery. In a recent survey of medical students, 66% reported an interest in global surgery, but 79% reported that global surgery is rarely addressed in their medical school curriculum [25]. This lack of education is reflected in the survey responses from students at a prominent medical school, where a minority (28%) of students correctly answered that trauma results in more deaths worldwide than obstetric complications or HIV/AIDS, malaria and tuberculosis combined. In this same survey, students perceived surgical care as cost-ineffective and only 3% of students believed that surgical care was a reliable indicator of a robust health care system [5]. Students in LMICs have expressed disinterest in choosing surgery as a career [26], and a recent survey of Ugandan medical students showed specialty choice was highly influenced by funding priorities, making surgery a less desirable choice [27].

Therefore, there is both a desire and a need for formalized educational opportunities in global surgery. To achieve advances in global surgical care, surgeons and non-surgeons alike must be educated regarding the challenges and societal benefits of investment in global surgery.

### Benefits and Difficulties of Interactive Group Learning

We observed the following results from our survey: 1) following the course, students tended to place a higher priority on surgery, and other non-communicable diseases in the global health agenda, 2) student self-assessed understanding of global health, global surgery and challenges faced by the underserved improved during the course, and 3) there was a stronger agreement after the course that surgery is an indispensable part of healthcare. Most students agreed that group learning assignments increased the value of course, enhanced their knowledge of the course material, and caused them to think more deeply about the topics.

Group learning, however, is only as good as the contributions of the members. Several students expressed that group learning was more time-consuming and it was difficult to coordinate their schedules outside of class to work on the assignments. Not surprising, it was a source of stress when other group members did not contribute equally to the work.

The students in the course were ethnically and racially diverse, and approximately one third were international students. This diversity enabled multiple viewpoints, and many students had first-hand experiences within the countries they had chosen to focus their assignments. This enabled group members to benefit from their teammate’s real-world perspectives. Although some students complained about the group assignments, we feel that the benefits outweigh the difficulties. Global challenges require multidisciplinary solutions, which by nature requires group cooperation. Western educational experiences often emphasize individual learning and assessment, but to maximize impact, students must learn to work in teams. To address the need for diverse representation of leaders in global surgery, we continue to modify our course on a regular basis to increasingly incorporate lectures and input from colleagues in LMICs and different fields of study.

To strengthen group dynamics, we have modified our course based on student feedback to include: 1) limit group size to three to four members, 2) encourage students to reserve a regular meeting time outside of class when they will commit to work on their assignments, 3) include an inter-group evaluation as part of the grading scheme, in which students are evaluated by their team members.

### Study Limitations

During the study period, students were enrolled in multiple courses regarding global health and other subjects. Therefore, pre- and post-course attitudes may have been influenced by other factors outside of the Global Surgical Care course. In addition, the small sample size limited the statistical power of the study and three students did not complete the post-course survey, which may have biased the results.

## Conclusion

We summarize an interactive, multidisciplinary course on global surgery which is designed to educate students on the importance of surgical care in the global health agenda and to teach methods for conducting global surgical research. At the conclusion of the course, students placed a higher priority on surgery in the global health agenda, indicated a greater understanding of challenges faced by the underserved in accessing surgical care, and agreed that surgery is an indispensable part of healthcare. The interactive nature of the course encouraged students to strengthen their skills in collaborative problem-solving and to learn from one another while thinking more deeply about the topics. It is our hope that similar courses may be developed in an inclusive worldwide model to educate surgeons and non-surgeons about the need and strategies for building healthy surgical systems worldwide.
